# Iterative versus non-iterative image reconstruction methods for sparse magnetic resonance imaging

**DOI:** 10.14312/2399-8172.2020-5

**Published:** 2020-08-03

**Authors:** Gengsheng L Zeng, Edward V DiBella

**Affiliations:** 1 Department of Radiology and Imaging Sciences, University of Utah, 729 Arapeen Drive, Salt Lake City, Utah, 84108, USA; 2 Department of Computer Science, Utah Valley University, 800 West University Parkway, Orem, UT 84058, USA

**Keywords:** tomographic image reconstruction, under-sampled measurements, fast MRI, analytics reconstruction

## Abstract

Magnetic resonance imaging (MRI) using under-sampled k-space data is a common method to shorten the imaging time. Iterative Bayesian algorithms are usually used for its image reconstruction. This paper compares an iterative Bayesian image reconstruction method that uses both spatial and temporal constraints and a non-iterative reconstruction algorithm that does not use temporal constraints. Three patient studies are performed. It is interesting to notice that the images reconstructed by the iterative Bayesian algorithm may introduce more bias than the non-iterative algorithm, even though the images provided by the iterative Bayesian algorithm look less noisy. The bias can be reduced by decreasing the influence of the temporal constraints.

## Introduction

Real-time or dynamic imaging in magnetic resonance imaging (MRI) require fast data acquisition, and the k-space data acquired is incomplete [1, 2]. When the data is incomplete, the conventional analytical image reconstruction methods usually produce severe aliasing artifacts. Using under-sampled data to reconstruct an image is sometimes referred to as a compressed sensing problem, which is commonly solved by constrained optimization. Iterative algorithms with constraints are able to produce images with less severe artifacts and with higher signal-to-noise ratios [3–8]. The iterative methods in image reconstruction commonly have an objective function that contains spatial and/or temporal constraints. For example, if the image is sparse, the sparseness can be enforced by using the L_0_ norm or L_1_ norm constraints. If the image is piecewise constant, the total variation (TV) norm constraint can be used. In MRI, if the k-space is scanned using a non-Cartesian sampling scheme, it is popular to first interpolate the non-Cartesian k-space measurements into the Cartesian grid before reconstruction [9–11].

Nowadays deep learning methods are popular in almost all areas of this modern world and they are well accepted in MRI image reconstruction and denoising [12–15]. Deep learning methods extract features from the training data, and their performance is training data dependent. It is not clear whether the selection of the training data affects the image bias, if the k-space data is incomplete and the image is reconstructed by a neural network.

Parallel imaging using multiple receiver coils in MRI is another way to achieve fast data acquisition [16–18]. Each coil can measure a different region in the k-space. The combined data from all coils is equivalent to a complete data set. This paper’s focus is on incomplete data image reconstruction. Therefore, the patient studies in this paper only use the (incomplete) sparse-data obtained from only one coil.

For image reconstruction with an incomplete data set, some a priori information is enforced to supplement the missing data. As a priori information, for example, one could assume that the image changes slowly in time and the image is piecewise constant. Bayesian image reconstruction methods in general present images with higher signal-to-noise ratios than the methods that do not use prior information.

There is one concern for all Bayesian methods. What happens if the a priori information is wrong? The Bayesian methods may thus cause bias. This paper compares an iterative Bayesian algorithm [19] and a non-iterative non-Bayesian algorithm [20] in terms of image bias using some MRI patient studies.

## Methods

### Iterative algorithm with spatio-temporal constraints

In this paper, we consider sparse radial k-space sampling in MRI, in which the number of views is not sufficient. The sparse-view data may cause streaking aliasing artifacts in the reconstructed images. Two algorithms are compared in this paper in terms of their performance for sparse-view MRI.

The iterative Bayesian algorithm used in the comparison studies here was taken from reference [19] and is briefly outlined as follows. The aim of the iterative algorithm is to minimize an objective function that consists of one data fidelity term and two Bayesian terms. The objective function, $C_{t}$, can be expressed as

(1) $$C_{t}=∥WFm-d{∥}_{2}^{2}+\alpha_{1}\sum_{i=1}^{N}\;{∥\nabla_{t}m_{i}∥}_{2}^{2}+\alpha_{2}\sum_{j=1}^{N}\;\sqrt{{\left(\nabla_{x}m_{j}\right)}^{2}+{\left(\nabla_{y}m_{j}\right)}^{2}+\varepsilon }$$

where m is the spatial domain image, F is the two-dimensional (2D) Fourier transform, W is the masking matrix depending on the k-space sampling scheme, N is the total number of image pixels, and ε is a small positive constant. The mathematical symbols $\nabla_{x},\;\nabla_{y},\;\nabla_{t}$ are the gradients in the x,y, the time (t) directions, respectively. The image is represented in the x−y Cartesian coordinate system. In the right-hand-side of Eq. (1), the first two terms use the $L_{2}$ norm, while the third term uses the total variation (TV) norm. The control parameters $\alpha_{1}$ and $\alpha_{2}$ determine the balance among these three norms in the objective function (or cost function). In our implementation, $\alpha_{1}=0.04,\;\alpha_{2}=$ 0.006,ε=0.00000001; these parameters were suggested in reference [19]. An iterative gradient descent algorithm is used to optimize the objective function, $C_{t}$, as defined in Eq. (1). The number of iterations is chosen to be 1000 as suggested in reference [19].

In this iterative algorithm, the sparse radial k-space measurements are first interpolated into a Cartesian grid, d. For a 2D image array m,d is a 2D complex matrix in the k-space. Since the k-space measurements are sparse, the majority of the elements in d is zero. Matrix W in Eq. (1) is a binary masking 2D matrix, with the same size as matrix d. The element of W is 1 if the corresponding element in d is measured. The element of W is 0 if the corresponding element in d is not measured. In Eq. (1), Fm denotes the 2D Fourier transform of the 2D image m. The notation WFm represents the element-by-element multiplication between the mask matrix W and the 2D Fourier transform, Fm, of the image m.

The image m reconstructed by this iterative algorithm is complex, which consists of a real part $m_{real}$ and an imaginary part $m_{imaginary}$. The last step in the image reconstruction algorithm is to convert the two 2D image arrays to the norm image on the element-by-element base. In other words, the final image is obtained as

(2) $$Final\;image=\sqrt{m_{real}^{2}+m_{imaginary}^{2}}$$

### Non-iterative algorithms without temporal constraint

The non-iterative algorithm used in our comparison studies is taken from reference [20] with a modification. This non-iterative algorithm does not use any constraints in the temporal direction. In other words, the images at different time frames are assumed to be independent. The main idea of this non-iterative algorithm is first to extend the sparse-view k-space measurements to more synthetic views in order to reduce the under-sampling aliasing artifacts and then to reconstruct the image with an analytical filtered backprojection (FBP) algorithm. The FBP algorithm is the most popular image reconstruction algorithm in computed tomography (CT).

Since the k-space data is complex, we process the real part and the imaginary part separately and obtain two images: the real part and the imaginary part. The final image is the norm image by combining the real-part image and the imaginary-image as indicated by Eq. (2). This is only aspect that this algorithm differs from the one developed in reference [20], where the complex k-space data was first converted into norm data by combining the real part and imaginary part. We believe that it is more proper to construct the real-part image and imaginary-part image first and then to form the norm image by combining these two images as the final step. The current method is mathematically equivalent to use the complex measurements directly for image reconstruction and convert the complex image to a norm image at the end.

**Figure 1:**
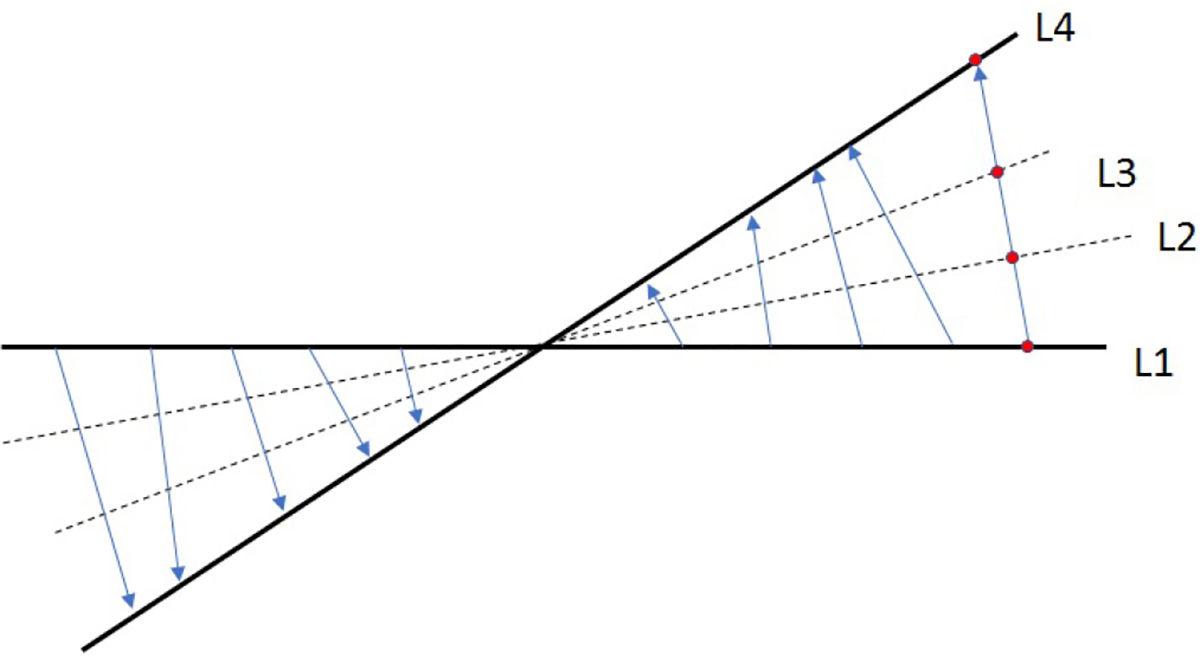
The values on line L1 are deformed on line L4 as indicated by the blue arrows.

The outline of the non-iterative algorithm is described as follows. Instead of interpolating the radial k-space measurements into a Cartesian grid, data on some unmeasured radial k-space lines is estimated by the deformation method. The deformation method is illustrated in Figure 1, where the two solid lines represent the locations where the measurements are available, and the two broken lines represent the locations where the measurements are not available and to be estimated. The values on line L1 are deformed to obtain the values on line L4, as indicated by the blue arrows in Figure 1, in the sense that for every value on L1 there is a corresponding close value on L4. The red dot on L1 and the red dot on L4 illustrate the corresponding values. Those blue arrows in Figure 1 form a deformation field. The name ‘deformation’ implies that L4 can be thought of being deformed from L1. The deformation method is to assign the ‘red dot’ value to lines L2 and L3 at the intersections with the deformation vector (i.e., the blue arrow). The deformation field (i.e., the blue arrows) can be estimated using a non-iterative algorithm. For more details of this deformation method, please consult reference [20].

In this paper, the sparse MRI data contains 24 k-space radial lines. The extended MRI data contains 72 k-space lines. Between every pair of measured lines, measurements on two new lines are estimated, as shown in Figure 1.

After the extended radial measurements are obtained, an analytic FBP is carried out as following steps for real part and imaginary part of the data separately:

Step1: Line-by-line filter the k-space measurements by a 1D transfer function

(3) $$H(\omega )=\frac{\left|\omega \right|}{1\;+\;\beta |\omega |}$$

where |ω| is the absolute value of the frequency, that is, the distance to the center of the k-space. The parameter β determines the level of regularization, as suggested by reference [21].

Step 2: Line-by-line take the 1D inverse Fourier transform.

Step 3: Perform backprojection.

Two images are obtained by application of the above three steps to the real-part and imaginary-part of the measurements. The final image is the norm image by combining these two images according to Eq. (2).

The transfer function (3) is a regularized version of the original ramp filter

(4) Ramp⁡(ω)=|ω|

as suggested by reference [21], aiming to find a minimum norm solution. This non-iterative algorithm can have multiple versions. Some of the versions are used in this paper for comparison studies:

Version 1: As described above, the input data for ‘Step 1’ is the extended 72-view measurements. The extended 72-view measurements are obtained by the deformation method from the 24-view raw measurements.

Version 2: The input data for ‘Step 1’ is the 24-view raw measurements. The three steps are the same as presented above.

Version 3: This version is almost the same as Version 2, except that the filter transfer function for ‘Step 1’ is the ramp filter given by Eq. (4), instead of the one in Eq. (3).

Version 4: This version is almost the same as Version 3, except that the input data for ‘Step 1’ is the 72-view ‘complete’ raw measurements. Note that these 72-vew measurements are directly acquired from the MRI scanner; they are not extended measurements from the 24-view raw measurements. The final image from Version 4 is treated as the gold standard for the results from other methods to compare.

### MRI patient data

Actual patient cardiac perfusion MRI data is used in this paper for comparison studies. All studies were approved by our Institutional Review Board (IRB). All participating patients were properly consented. Patient identifiable information was removed before transferring to our research computers for processing.

Data acquisition was performed using a Siemens 3T Trio scanner. A phased array of multiple of coils was used during data acquisition; however, data from only one selected coil was used in image reconstruction. The scanner parameters for the radial acquisition were TR = 2.6 ms, TE = 1.1 ms, flip angle = 12°, Gd dose = 0.03 mmol/kg, and slice thickness = 6 mm. Reconstruction pixel size was 1.8 × 1.8 mm^2^. Each image was acquired in a 62 ms readout. The acquisition matrix size for an image frame was 256 × 72, and 75 sequential images were obtained at 75 different times. At each time frame, the k-space is sampled with 72 uniformly spaced radial lines over an angular range of 180°.

For the comparison purposes of this paper, at each time frame we uniformly under-sampled the 72 views into 24 views and call such obtained data as 24-view raw data. The FBP reconstructed images with 72 views were treated as the gold standard. The image discrepancy between the gold standard and other images are evaluated in terms of root-mean-square-error (RMSE). A median filter is applied to all images before the RMSEs were measured and displayed.

## Results

**Figure 2:**
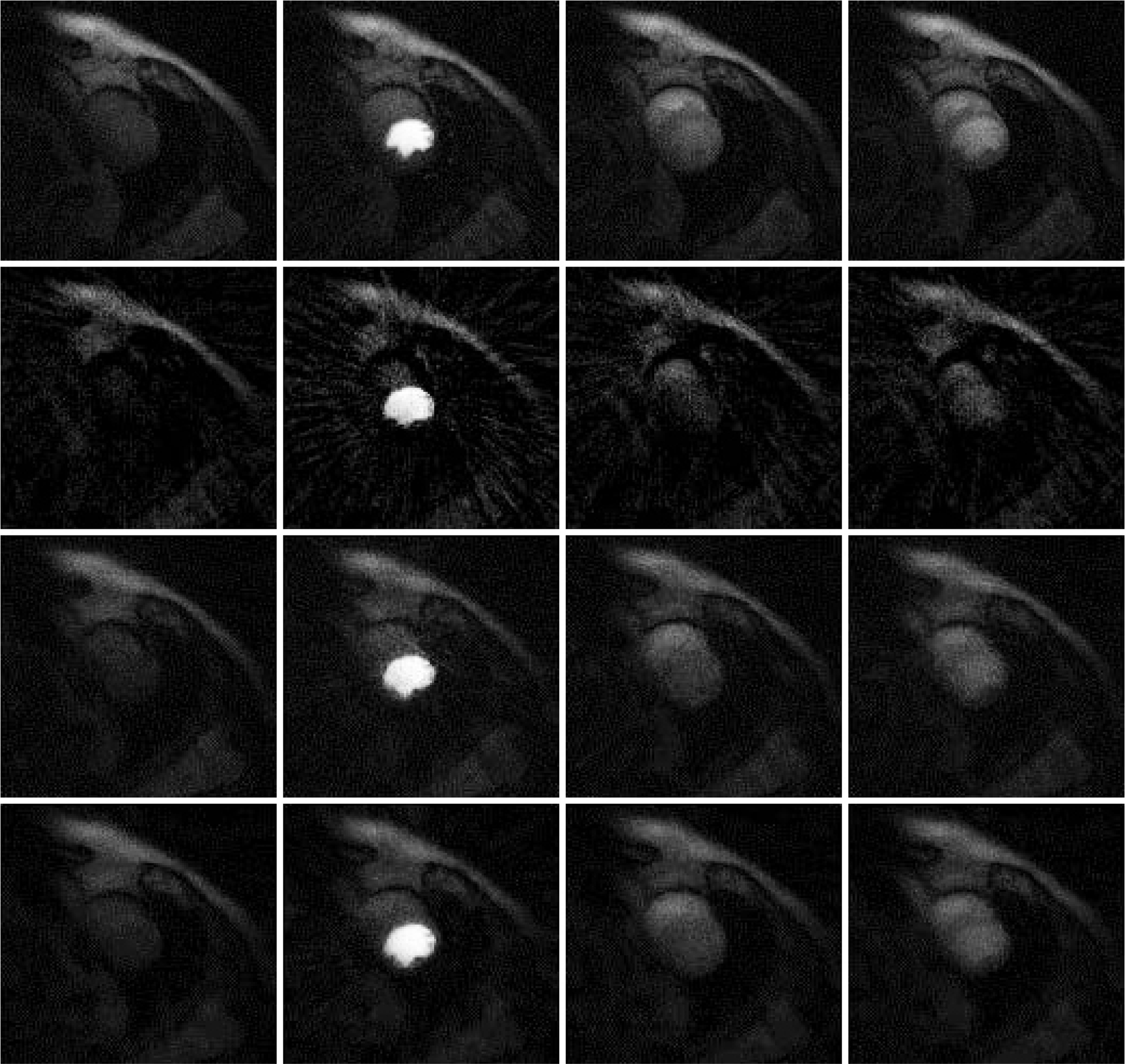
Images reconstructed from Patient 1 data. Column 1: Time frame 6, Column 2: Time frame 21, Column 3: Time frame 36, and Column 4: Time frame 51. Row 1: Gold standard FBP with 72 views (Version 4), Row 2: 24-view raw FBP reconstruction (Version 3), Row 3: 24-view extension to 72-view, then FBP with β = 1 (Version 1), and Row 4: iterative Bayesian with α_1_ = 0.04.

**Figure 3:**
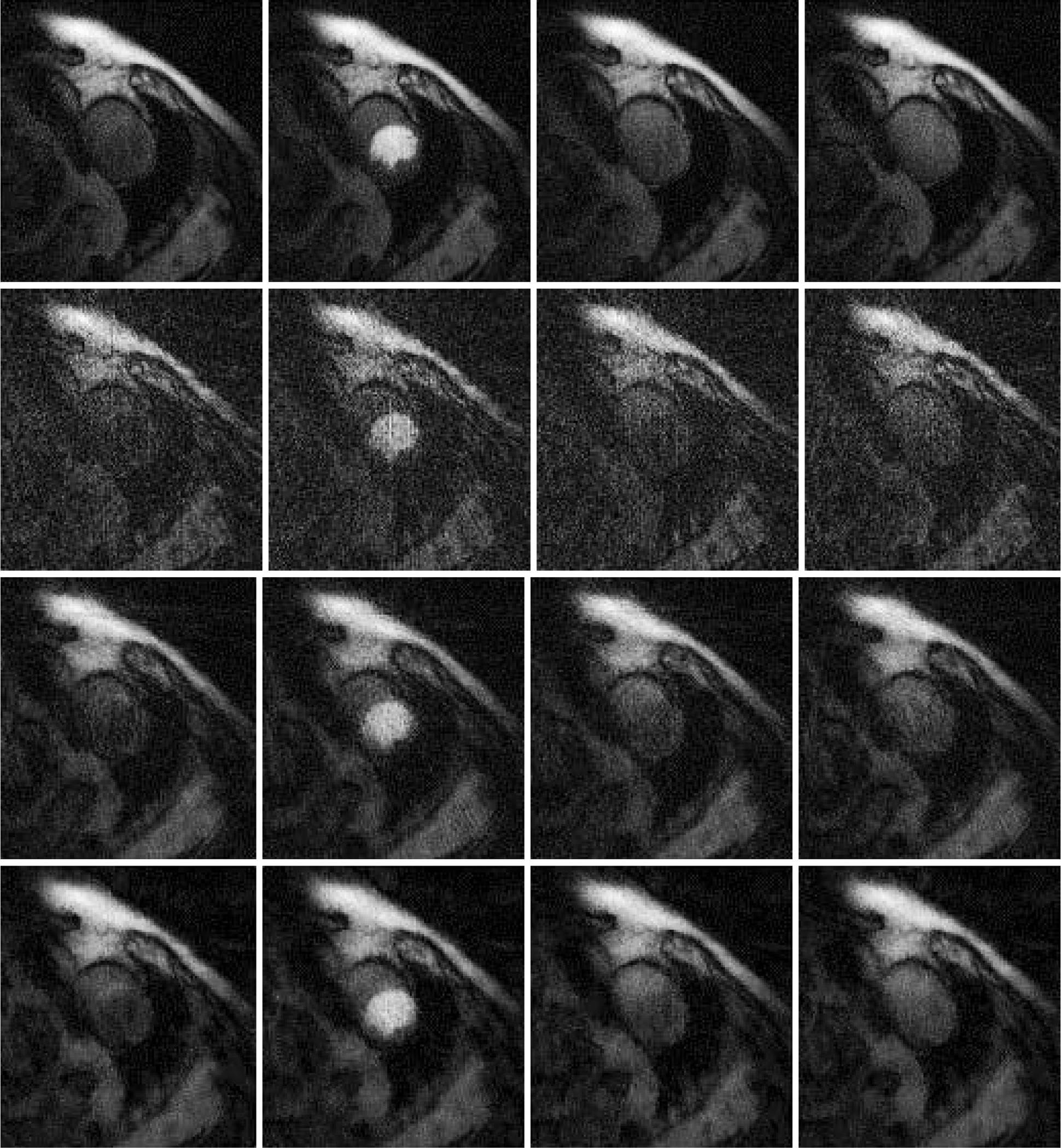
Images reconstructed from Patient 2 data. Column 1: Time frame 6, Column 2: Time frame 21, Column 3: Time frame 36, and Column 4: Time frame 51. Row 1: Gold standard FBP with 72 views (Version 4), Row 2: 24-view raw FBP reconstruction (Version 3), Row 3: 24-view extension to 72-view, then FBP with β = 1 (Version 1), and Row 4: iterative Bayesian with α_1_ = 0.04.

**Figure 4:**
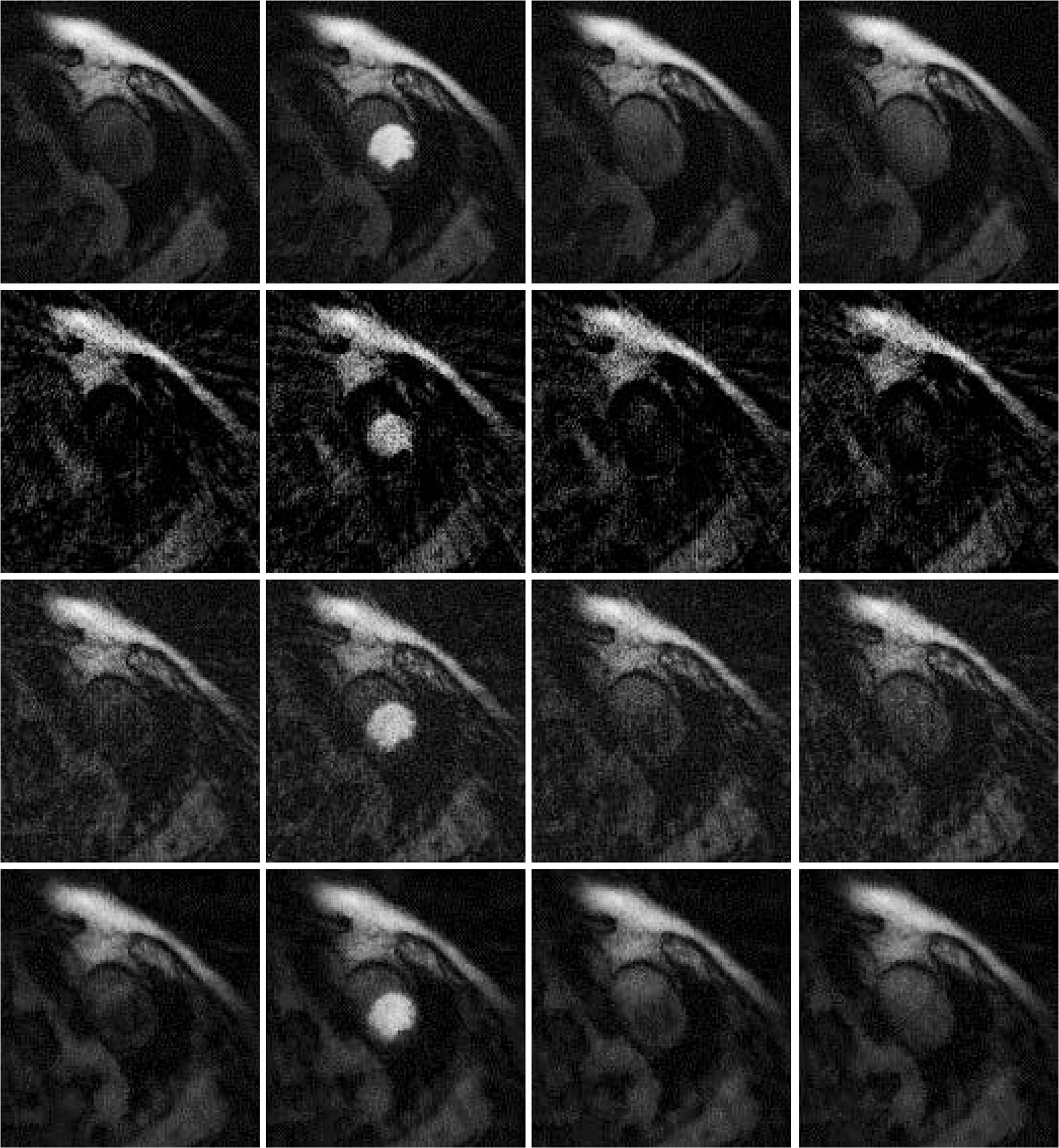
Images reconstructed from Patient 3 data. Column 1: Time frame 6, Column 2: Time frame 21, Column 3: Time frame 36, and Column 4: Time frame 51. Row 1: Gold standard FBP with 72 views (Version 4), Row 2: 24-view raw FBP reconstruction (Version 3), Row 3: 24-view extension to 72-view, then FBP with β = 1 (Version 1), and Row 4: iterative Bayesian with α_1_ = 0.04.

Results are shown in Figures 2, 3 and 4, for patient 1, patient 2 and patient 3, respectively. For each patient, there are 5 sets of reconstructed images by the iterative Bayesian reconstruction algorithm and by the 3 versions of the non-iterative reconstruction methodology. Each set consists of 75 sequential images. There would be too many images to show if all images were displayed. To make the paper more readable, 4 (instead of 75) images are shown for each set. Table 1 lists the root-mean-square-errors (RMSEs) of images deviate from the gold standard.

It is clear from Table 1 to observe that directly using the 24-view raw data gives the worst performance. The iterative algorithm and the non-iterative algorithm have similar performance, with the non-iterative algorithm’s results being slightly better.

All these algorithms have some parameters to adjust. It is interesting to observe that when the parameter α_1_ in the objective function (1) is reduced from 0.04 to 0.004, the RMSE is reduced. We conjecture that the RMSE for the iterative algorithm is caused by the bias. Reducing the influence of the temporal constraint in the objective function (1) reduces the bias. A Bayesian constraint can help the image look better, but at the same time, the Bayesian constraint can change the image value.

**Figure 5:**
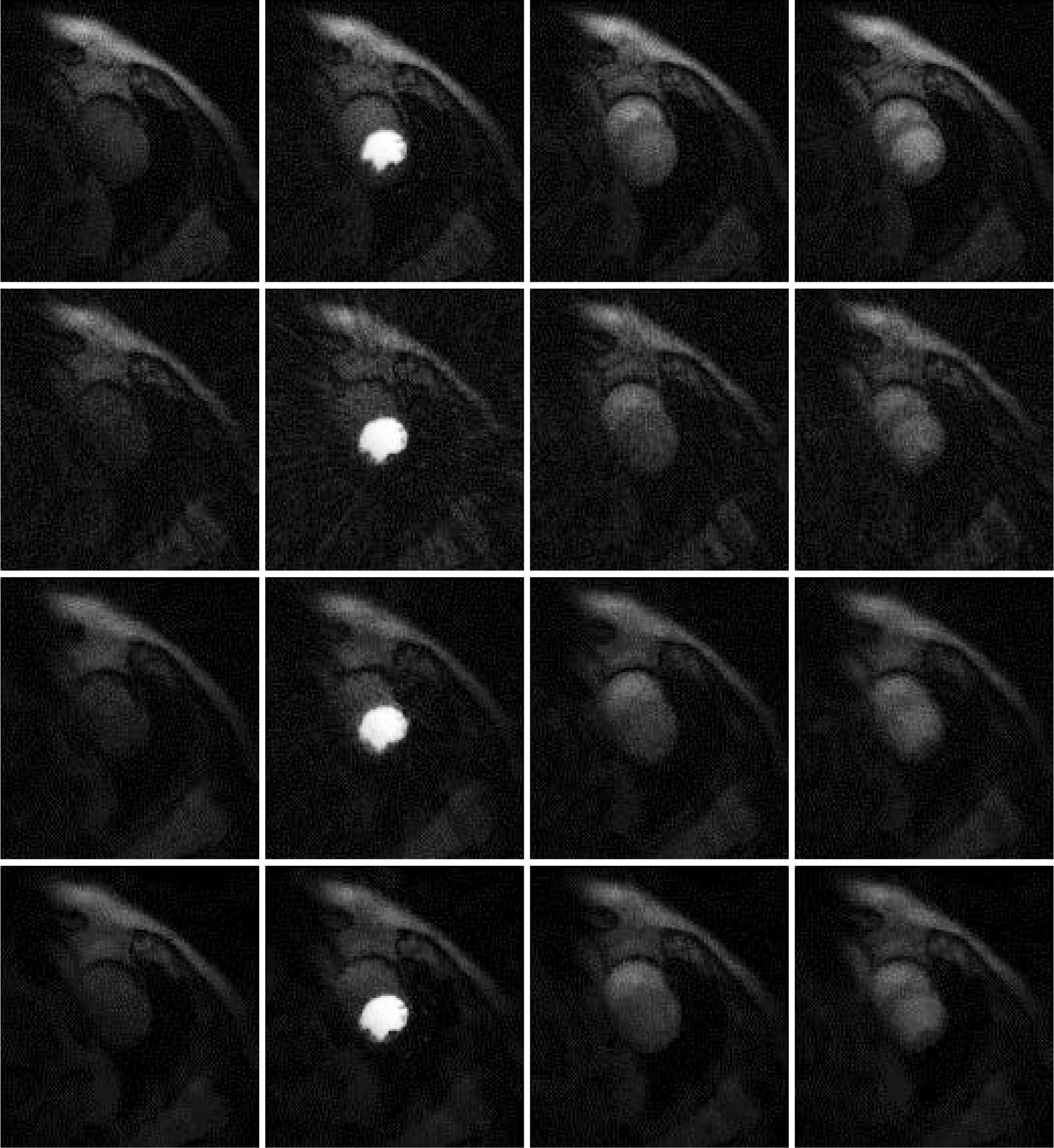
Images reconstructed from Patient 1 data. Column 1: Time frame 6, Column 2: Time frame 21, Column 3: Time frame 36, and Column 4: Time frame 51. Row 1: Gold standard FBP with 72 views (Version 4), Row 2: 24-view raw FBP reconstruction (Version 2), Row 3: 24-view extension to 72-view, then FBP with β = 2 (Version 1), and Row 4: iterative Bayesian with α_1_ = 0.004.

For the non-iterative algorithm, a regularization factor β|ω| is introduced to encourage a minimum norm solution. When β = 0, there is no regularization. When β is large, the regularization is heavily enforced. In Figures 5~7 and in Table 2, some other cases are shown. One case is the iterative reconstruction with a smaller α_1_ = 0.004. Another case is the non-iterative reconstruction with heavier regularization using β = 2. The third case is the non-iterative reconstruction with β = 1 and with 24-view raw data, where the 24 views are not extended to 72 views.

From Figures 5~7 and Table 2, we observe the following: For the iterative algorithm, reducing the temporal constraint helps reducing the bias. Increasing the regularization in the non-iterative algorithm helps to reduce the noise. If the 24-view data is not extended to 72-data, the regularization helps denoising, but extension to 72-view data can further reduce some artifacts.

The structural similarity index measure (SSIM) is another well accepted figure-of-merit to find the similarity of two images; the definition details of SSIM are given in [22]. The counterparts of Tables 1 and 2 are Tables 3 and 4, respectively. The results of Tables 3 and 4 are consistent with those in Tables 1 and 2, indicating the superiority of the proposed non-iterative algorithm. The optimal value for RMSE is 0, and the optimal value for SSIM is 1.

## Discussion

The iterative algorithm uses the TV norm for regularization in the image spatial domain. The TV norm also in favor of piecewise constant images and may cause staircase artifacts when encouraging sharp edges. On the other hand, the non-iterative algorithm uses a lowpass filter 1/(1+β|ω|) to regularize the image by controlling the noise. This lowpass filter was derived by encouraging a minimum norm solution [21]. We observe from Table 2 that by increasing β value from 1 to 2 the noise is reduced and the RMSE is also reduced. However, the SSIM is reduced for one patient and increases for the other two patients. Therefore, using a lowpass filter alone is not effective in reducing the aliasing artifacts caused by under-sampled data. The non-iterative deformation method is used to estimate some unmeasured data. Comparing Table 1 with Table 2 and Table 3 with Table 4, the extension of the 24-view data to 72-view data actually improves both the RMSE and SSIM. In other worlds, Version 1 is better than Version 2.

We must point out that the extended 72-view data is different from the actually measured 72-view data and contains estimation errors. Of course, there is no substitute for the real data. If the real data is not available, the estimated data is the second choice. There are numerous ways to estimate the unmeasured data. The iterative algorithm in this paper estimates the missing data from the measured data in different time frames adjacent to the current time, while the non-iterative algorithm in this paper estimates the missing data from the measured data at the current time.

## Conclusions

In dynamic MRI imaging, time-activity curves are used to estimate tissue kinetic parameters, which have wide applications in clinical diagnoses. Accurate kinetic parameters depend on the unbiased time-activity curves that are extracted from dynamic images. The dynamic images are reconstructed using sparse k-space data.

Two sparse-data image reconstruction methods are compared into this paper: an iterative Bayesian method that uses a temporal constraint and a non-iterative method that does not have a temporal constraint. According to the root-mean-square-error (RMSE) analysis and structural similarity index measure (SSIM) analysis, these two methods give similar accuracies as shown in Tables 1~4. The non-iterative method is much faster than the iterative method. In the iterative reconstruction algorithm, the 24-view sparse-data is intrinsically extended to 72-view data by using the temporal constraint as shown in the objective function (1). The ‘assisting’ 48-view data is most likely different form the actual measured data. The influence of the ‘assisting’ 48-view data may bring in image bias. This bias is the price we pay to reduce the data under-sampling artifacts. After reducing the weighting parameter α_1_ from 0.04 to 0.004 in the iterative algorithm, the bias is reduced and the RMSE and SSIM are improved.

Our take-home message is that Bayesian constraints may make the images look better, but they may cause more image bias, deviating from the true solution. We must be aware of the tradeoff between looking good and accuracy.

Our future research includes investigations of deep learning methods for sparse-data MRI, in which the unmeasured data is supplemented by training data of other patients. The deep learning methods are effective but are not well understood, in terms of how the a priori information is presented and used. We will focus on whether the deep learning methods may introduce image bias when the k-space is not fully sampled.

## Figures and Tables

**Figure 6:**
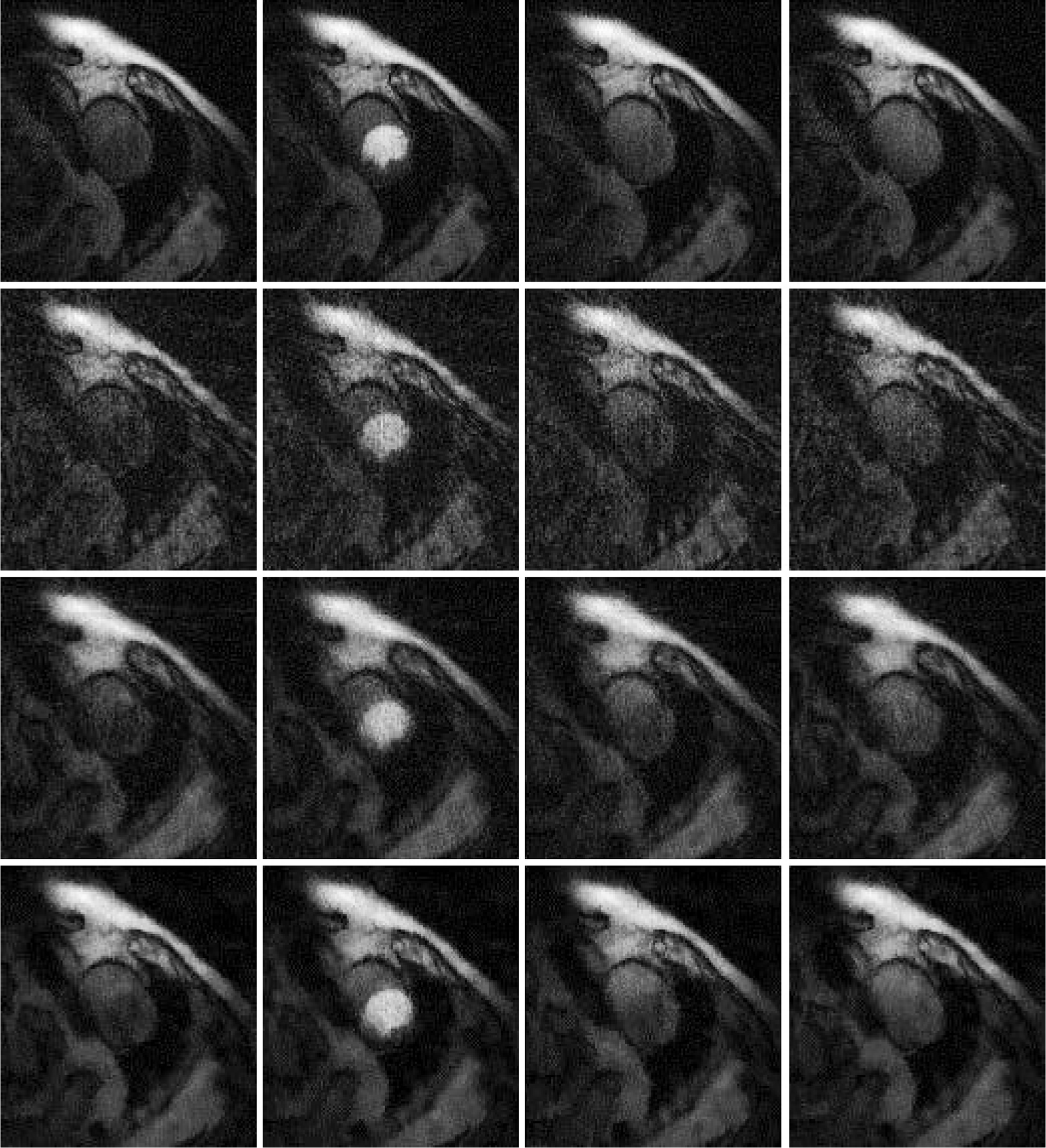
Images reconstructed from Patient 2 data. Column 1: Time frame 6, Column 2: Time frame 21, Column 3: Time frame 36, and Column 4: Time frame 51. Row 1: Gold standard FBP with 72 views (Version 4), Row 2: 24-view raw FBP reconstruction (Version 2), Row 3: 24-view extension to 72-view, then FBP with β = 2 (Version 1), and Row 4: iterative Bayesian with α_1_ = 0.004.

**Figure 7:**
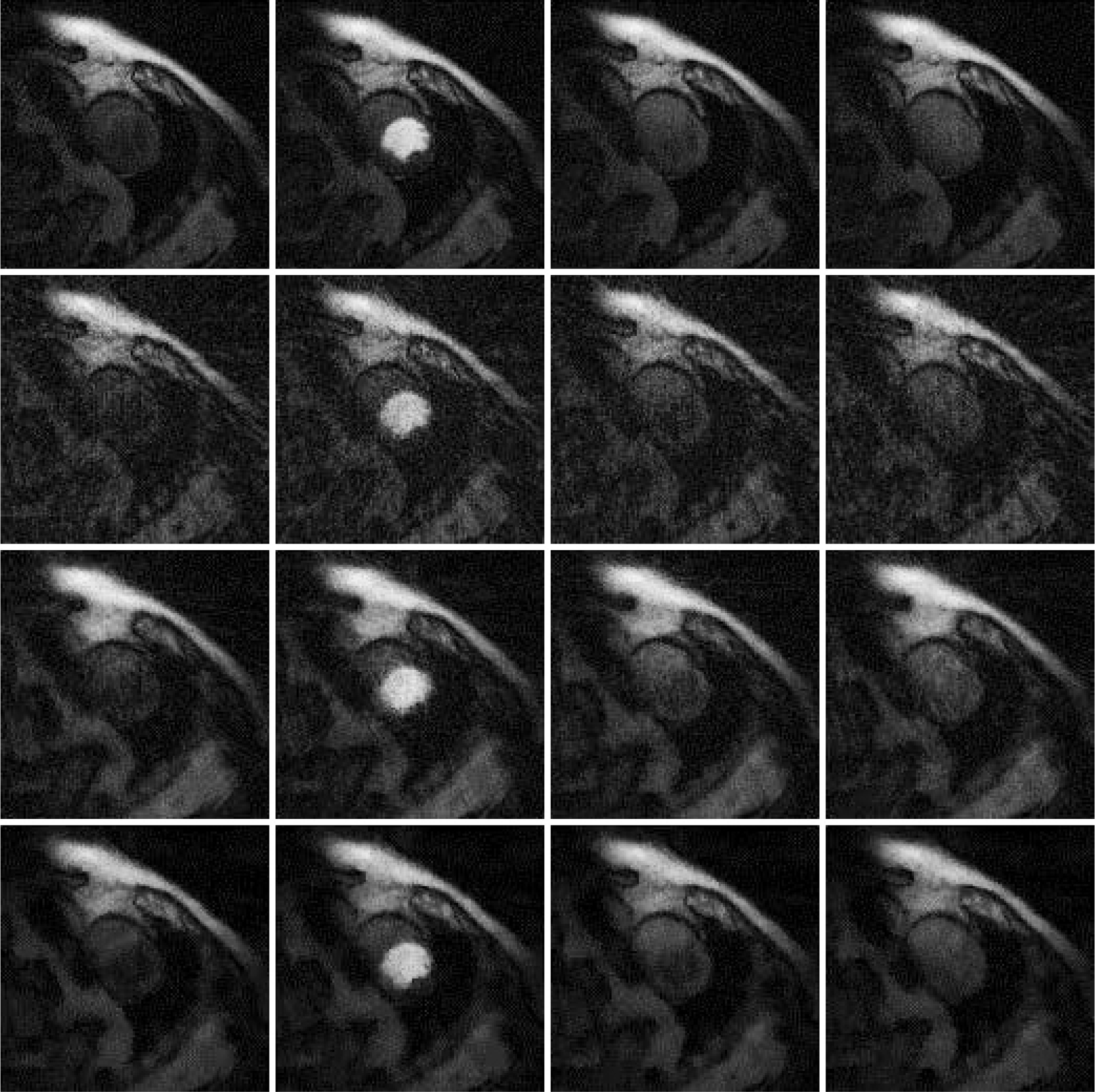
Images reconstructed from Patient 3 data. Column 1: Time frame 6, Column 2: Time frame 21, Column 3: Time frame 36, and Column 4: Time frame 51. Row 1: Gold standard FBP with 72 views (Version 4), Row 2: 24-view raw FBP reconstruction (Version 2), Row 3: 24-view extension to 72-view, then FBP with β = 2 (Version 1), and Row 4: iterative Bayesian with α_1_ = 0.004.

**Table 1:** RMSE for various reconstruction methods for three patient studies (using an initial set of parameters). Closer to 0 is better.

	72-view non-iterative (gold standard) β = 0	24-view non-iterative (raw reconstruction) β = 0	24-view → 72-view, non-iterative, without temporal constraint β = 1	24-view, iterative Bayesian, with temporal constraint α1 = 0.04

Patient 1	0	6.7675	3.6778	4.1719
Patient 2	0	10.957	5.0849	5.3663
Patient 3	0	10.747	4.9476	5.3545

**Table 2:** RMSE for various reconstruction methods for three patient studies (using another set of parameters). Closer to 0 is better.

	72-view non-iterative (gold standard) β = 0	24-view non-iterative (raw reconstruction) β = 1	24-view → 72-view, non-iterative, without temporal constraint β = 2	24-view, iterative Bayesian, with temporal constraint α1 = 0.004

Patient 1	0	4.2129	3.4196	3.9046
Patient 2	0	6.4403	4.6963	4.7018
Patient 3	0	6.2731	4.5616	4.6803

**Table 3:** SSIM for various reconstruction methods for three patient studies (using an initial set of parameters). Closer to 1 is better.

	72-view non-iterative (gold standard) β = 0	24-view non-iterative (raw reconstruction) β = 0	24-view → 72-view, non-iterative, without temporal constraint β = 1	24-view, iterative Bayesian, with temporal constraint α1 = 0.04

Patient 1	1	0.999635875413527	0.999848361889880	0.999807461269317
Patient 2	1	0.999413359233490	0.999804906454934	0.999768375404101
Patient 3	1	0.999421484562476	0.999814647345931	0.999769234887647

**Table 4:** SSIM for various reconstruction methods for three patient studies (using another set of parameters). Closer to 1 is better.

	72-view non-iterative (gold standard) β = 0	24-view non-iterative (raw reconstruction) β = 1	24-view → 72-view, non-iterative, without temporal constraint β = 2	24-view, iterative Bayesian, with temporal constraint α1 = 0.004

Patient 1	1	0.999800444659817	0.999849666451081	0.999819971016024
Patient 2	1	0.999697329429499	0.999803495341621	0.999799850215280
Patient 3	1	0.999705810078143	0.999813456704611	0.999801863895034

## A list of abbreviations

FBP: Filtered backprojection
MRI: Magnetic Resonance Imaging
1D: One dimensional
RMSE: Root mean square error
SVD: Singular value decomposition
TV: Total variation
